# Abundance, Diet and Foraging of Galápagos Barn Owls (*Tyto furcata punctatissima*)

**DOI:** 10.3390/ani15152283

**Published:** 2025-08-05

**Authors:** Hermann Wagner, Sebastian Cruz, Gustavo Jiménez-Uzcátegui, Katherine Albán, Galo Quezada, Paolo Piedrahita

**Affiliations:** 1 Institute of Biology II, RWTH Aachen University, Worringerweg 3, D-52074 Aachen, Germany; 2 American Bird Conservancy, Puerto Ayora 200102, Ecuador; sebastiancruz@abcbirds.org; 3 Charles Darwin Research Station, Charles Darwin Foundation, Galápagos, Puerto Ayora 200102, Ecuador; gustavo.jimenez@fcdarwin.org.ec; 4 Facultad de Ciencias de la Vida, ESPOL, Guayaquil 090902, Ecuador; katherine.alban.morales@univie.ac.at (K.A.); ppiedra@espol.edu.ec (P.P.); 5 Galapagos National Park Directorate, Santa Cruz, Puerto Ayora 200102, Ecuador; gquezada@galapagos.gob.ec

**Keywords:** ecosystem service, mouse, foraging, rat, data logger, pellet, arthropod, rodent, territory, population density

## Abstract

We studied Galápagos barn owls on Santa Cruz Island in the Galápagos Archipelago. We collected and analyzed pellets to determine diet composition. Barn-owl diet consisted—in terms of biomass—of ~89% rodents and ~10% insects. Bird remains occurred in 1% of the pellets. Foraging was studied with data loggers, a method not previously applied to the study of Galápagos barn owls. Owls rested during the day in natural and human-built roosts such as lava holes, trees, or huts. Night-time foraging was characterized by periods during which the bird moved and periods during which the bird stayed within one place, with the latter amounting to ~56% of the time away from the day roost. Birds began foraging shortly after sunset and returned to their day roost before sunrise. The duration of foraging was approximately 11 h per night. Foraging areas were small (median value: 0.28 km^2^). Although our data demonstrate a continued presence of the subspecies, we regard the situation for this subspecies as labile, as multiple threats, such as road kills, poisoning, and intentional killing by farmers, have increased recently, and suggest the development of a management plan to improve its conservation.

## Simple Summary

Barn owls are one of two owl subspecies that inhabit the Galápagos Islands. Since data on these birds have been lacking since the 1980s, we initiated a project in 2016 to study the abundance, prey, and foraging behavior of barn owls on Santa Cruz, the island with the largest human population in the archipelago. We observed barn owls both in agriculture-dominated and remote, natural areas. The observed foraging areas of Galápagos barn owls were much smaller than those observed in Europe or Argentina. The diet of the Galápagos barn owls consisted of ~89% rats and mice and ~10% arthropods. Bird bones were detected in 1% of the pellets. Barn owls provide ecosystem services by controlling invasive rodents. Threats to barn owls come from car traffic, poisoning, and intentional harm by humans who believe owls hunt chickens or symbolize misfortune or death. Galápagos barn owls are listed as endangered and warrant increased protection due to the ecosystem services they provide and the anthropogenic threats they face. A management plan and an education program could improve the status of the barn owl in this unique world-famous heritage site.

## 1. Introduction

The Galápagos Islands are a biodiversity hotspot and a World Heritage Site. Their fauna and flora require special attention in a changing world. Two endemic subspecies of owls inhabit the Galápagos Islands: the Galápagos barn owl (*Tyto furcata punctatissima*) and the Galápagos short-eared owl (*Asio flammeus galapagoensis*) [1,2,3]. Both subspecies have been historically understudied among the avifauna of the Galápagos Islands, although recent data on the short-eared owl are available [4]. By contrast, the last systematic study of Galápagos barn owls was conducted more than 40 years ago [5]. Given the lack of more recent data, it seems timely to re-examine the status of barn owls in the Galápagos Islands. This is important because the Galápagos barn owl is recognized as a distinct subspecies of the American barn owl (*Tyto furcata*) [6,7]. Compared to the continental populations, Galápagos barn owls are markedly smaller, averaging around 250 g in body mass, whereas North American barn owls weigh approximately 500 g [8]. A note for clarification: the term “barn owl” hereafter shall refer to the Galápagos barn owl, unless stated otherwise.

All barn owl species are nocturnal hunters that rely on excellent hearing and low-light vision to detect prey [9,10,11]. In addition, owls have developed silent flight [12,13]. Moreover, barn owls possess specialized cognitive capabilities, such as visual-search behavior [14,15,16]. Barn owls frequently inhabit areas near human settlements, where they rest in inconspicuous roosts [11]. In Europe, these birds typically start their nocturnal activity about 30 min after sunset and typically return to their day roosts about 30 min before sunrise ([11], own observations by HW). De Groot [5] reported that Galápagos owls spent 72% of their hunting time on perches. Owl diet may be faithfully reconstructed from pellets [11].

The Galápagos barn owl colonized the Islands long before human arrival. The islands’ volcanic landscape provided natural cavities, such as lava tunnels, which served as roosting and nesting sites. Following human settlement, barn owls began to use artificial structures, similar to their behavior in Europe and North America. Barn owls are considered synanthropes, species that have adapted to live in close proximity to humans and benefit from the environmental modifications [17]. Early evidence of the presence of barn owls on the Galápagos Islands comes from the Beagle expedition [18] and from skins stored, for example, at the Natural History Museum in San Francisco (USA) or the Charles Darwin Research Station in Puerto Ayora. A German expedition in the 1960s provided information on the diet of barn owls on the islands [19]. In 1979/1980, de Groot [5] studied the origin, status, and ecology of Galápagos owls.

Since the late 1960s, the human population in the Galápagos Islands has increased from a few thousand to approximately 28,500 in 2022 (Table 5 in [20]). This rapid growth has been accompanied by increased road construction, deforestation, and a rise in vehicle traffic. Cars were introduced in the 1960s, and the number of registered cars rose to more than 2900 by 2018 [21,22,23]. Climate change has also begun to impact the archipelago, further increasing the pressures on nature just mentioned [24].

We regard it important to study this endemic subspecies to understand how these environmental changes affect its population and behavior and provide data for the development of a conservation plan. Therefore, we launched the project “Conservation of Galápagos Owls: Numbers, Diet, Foraging Ranges, and the Influence of Owls on Populations of Endemic and Introduced Mammals and Birds” in 2016. Since little data had been known about barn owls on Galapagos for 40 years, our goal was to collect a broad variety of data. We approached this goal by collecting pellets, by obtaining foraging data with loggers, a method that had not been previously applied to the study of Galápagos barn owls, and by covering a large area with our excursions to obtain a general overview of barn owl abundance in the agricultural zone of Santa Cruz. In this paper, we report data on diet, foraging, and abundance. Other aspects, such as wing morphology and feather condition, and their possible influence on survival, were analyzed and published separately [8,25].

## 2. Materials and Methods

**Figure 1:**
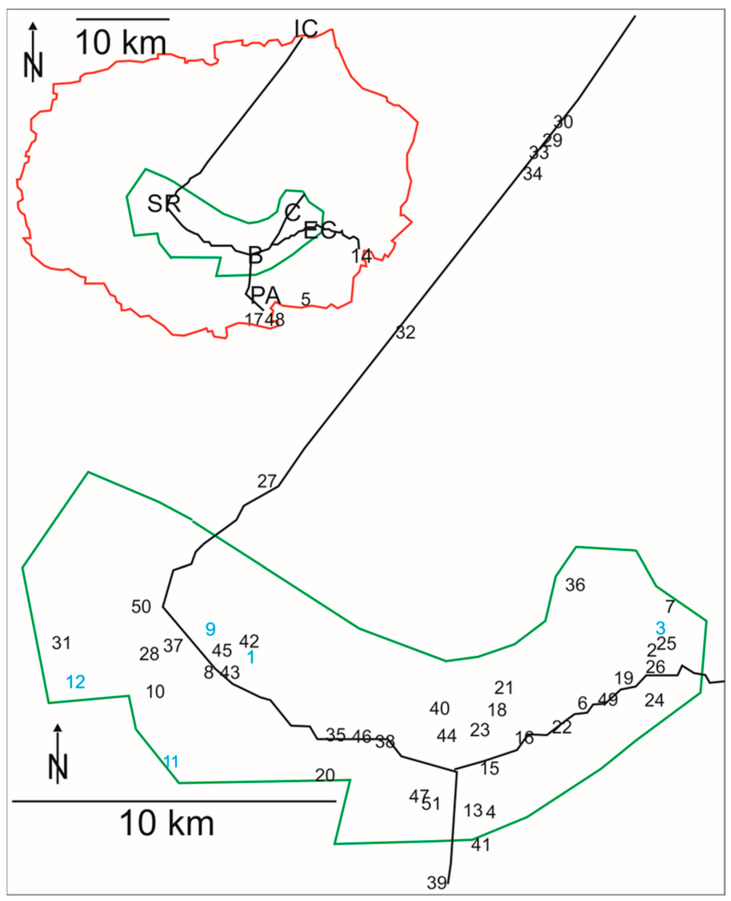
Locations where we encountered signs of owls on Santa Cruz. The locations are numbered consecutively from 1 to 51. The inset shows the whole island. Numbers in turquoise indicate locations where logger data were obtained. Letters in the inset indicate important locations: PA: Puerto Ayora, B: Bellavista, EC: El Cascajo, SR: Santa Rosa, C: Camote, IC: Itabaca Channel, red: borders of the island, green: border of agricultural area, black: important roads. Further details in Table S1.

This study is based on data from the Galápagos barn owl (*Tyto furcata punctatissima*). Data were collected under permits issued by the Galápagos National Park Directorate (GNPD) (PC-19-16; PC-22-17; PC-28-18; PC-73-19). To advertise our project, we distributed flyers that informed locals about owls and asked for information on owl sightings. The study was initially designed as a systematic study covering all islands and areas where barn owls had been observed in the past. However, it was realized early in the field that this goal was unrealistic. Therefore, we concentrated data collection on Santa Cruz, focusing on accessible sites like roads, settlements, and farms in the agricultural area. This restriction was driven by the fact that the work with native fauna in Galápagos is regulated by strict environmental rules, which limit the number of sites, sampling periods and handling of individuals. In this way, data came from three different sources: (1) We (PP and HW) visited Galápagos in the hot season (January–May) of 2016 and 2017 and in the cold season (June–December) of 2018 and 2019. Data collected include observations during excursions in the field, mainly to farms and settlements. Most birds were detected at their roosting sites during the day. Excursions were planned and conducted together with staff from GNPD. Information provided by residents helped to find owl roosts. Excursions covered a large area within the agricultural zone (Figure 1). In total, we went on 121 excursions (36 in 2016; 30 in 2017; 34 in 2018; 21 in 2019) between 2016 and 2019. During the excursions we looked for signs of owls: the presence of birds, pellets, feathers, and eggs. After 2019, residents continued to send reports of owl observations, which are also included here. The most recent data are from 2022. (2) We also include barn-owl data collected during a road project initiated by one of the authors (GJU). This project was designed as a line count along the road from Puerto Ayora to the Itabaca Channel. (3) Some data—especially pellets–were collected by rangers from the GNPD, and these samples are also included here.

Pellets were collected wherever we found them, provided they were intact. Disintegrated pellets were not collected. Pellets were found at numerous sites on Santa Cruz Island (Figure 1, Table S1). Locations where pellets were found covered many places within the agricultural zone. In total, 523 pellets were used for analysis. Analysis was conducted at the Escuela Superior Politécnica del Litoral (ESPOL) (Guayaquil, Ecuador). The collections of the Charles Darwin Foundation (CDF) were available for the identification of remains in the pellets. Barn owl pellets from Santa Cruz could be easily discriminated from those of the Galápagos short-eared owl. The pellets of the Galápagos barn owl were smaller than those of the Galápagos short-eared owl. The size of the barn-owl pellets was between 2 and 4 cm in length, while short-eared owl pellets typically had a length of more than 5 cm. Barn-owl pellets were typically darker than short-eared owl pellets and more compact. While the Galápagos hawk (*Buteo galapagoensis*) also produces pellets, its pellets are different from those of the owls, because hawks can better digest bones. Furthermore, the Galápagos hawk is very rare on Santa Cruz and does not cast pellets in human structures or lava holes.

We studied the foraging movements of barn owls with GIPSY-5 data loggers (Technosmart, Rome, Italy). Birds were caught, physically examined to check their health status and size, ringed, fitted with a backpack containing the GPS logger, and released. The logger data obtained just after the release of the birds suggested that the birds rested in the day roosts as they would normally do. The weight of this backpack was ~9–10 g, i.e., intended to amount to less than 5% of the body mass of the birds (227–290 g) [8]. After 1 to 8 days, the birds were re-caught, and the data were read out or the logger was removed. Similar methods have been used successfully before [26]. The re-caught birds were physically examined a second time. This time we specifically checked whether the logger had hurt the birds or whether the birds had lost weight. Our examinations suggested that this was not the case. Moreover, we detected signs that the birds had been eating and had cast pellets. The fixation of the logger was carried out according to a protocol authorized by GNPD.

Loggers were programmed prior to deployment, with recording intervals set to collect frequent data at night (1 datum/20 s to 1 datum/3 min) and fewer records during the day (typically 1 per hour). These intervals were chosen to maximize battery power and duration. The GPS trackers were initially tested for spatial resolution both in Aachen and on the Galapagos. Spatial resolution depended on the availability of satellites, i.e., of how many satellites a logger could resister at a given time. We found that spatial resolution, measured as precision, could be as low as a few meters, but depended on the specific environmental situation (see also Section 3.3.1). Data have been uploaded to Movebank (Galápagos barn owl project: https://www.movebank.org/cms/webapp?gwt_fragment=page=search_map, accessed on 29 July 2025).

The following parameters were extracted from the data recorded by the logger:

Foraging area: We estimated the foraging area with a variant of the minimum convex polygon technique, the smallest polygon in which no internal angle exceeds 180 degrees and which contains (nearly) all data points [27]. We excluded such data points that were clearly outliers, i.e., single points that were far away from the bulk of the other points (for examples see Section 3.3.1).

GPS data were further analyzed with respect to the start and the end of the daily excursions, the foraging duration, the stop-over points, and stop-over durations during night-time foraging.

Start time: Time when the owls left the day roost in the evening. During the day, the owls were often hiding in places where the data logger did not detect a signal. The logger was programmed to check after a preprogrammed time (typically 20 min) for a signal. In those cases, in which the logger did not detect a signal before the owl left the day roost, the start time was the mean time between the last check before a signal was obtained and the first data point when a signal was obtained. In this way, temporal resolution was typically 10 min.

End time: Time when the owls returned to the day roost in the morning. Typically, the end time was the mean time between the last data point during the outing of the owl and the first time when the signal was lost. Temporal resolution was typically one recording interval or 3 min.

Foraging duration: The time difference between the end time and the start time.

Stop-over points: During night-time foraging the owls had phases where they moved around and periods where they were stationary. A stop-over point was defined as a location when the owl did not exhibit a clear movement for at least 5 data points, typically 12 min. This lower limit was chosen to avoid mis-judging. However, by applying this lower limit very short stop-over points (<12 min) might have been missed. The mean location during a stopover was calculated as the average of all data points recorded during the stopover.

Stop-over duration: The time difference between the start and end of a stop-over.

Percent of non-motion: The sum of the durations of all stop-overs during a night divided by foraging duration and expressed in percentage (%) of foraging duration.

Note that the time of day is written in 24-h time format. A Mann-Whitney U-test was used to check for differences between males and females (https://www.socscistatistics.com/tests/mannwhitney/default2.aspx, accessed on 16 July 2025).

## 3. Results

In total, we report signs of barn owls from 51 locations during the time from 2016 to 2022 (Figure 1, Table S1). In the following sections, we shall first show the locations visited. Then we shall present data on diet, before we turn to foraging. We end with a short section on owl abundance.

### 3.1. Locations Visited and Locations Where Signs of Barn Owls Were Encountered

Line counts along the road from Puerto Ayora to the Itabaca channel during the day resulted in the detection of several dead birds within the agricultural area (Figure 1 and Table S1: 35, 46) and north of this area (Figure 1 and Table S1: 33, 34). These birds were hit and killed by cars. During line counts on this road in the night, birds called in several places (Figure 1 and Table S1: 27, 29, 30, 32). Likewise, during several line counts on the road to El Garrapatero (14 in Figure 1) barn owls were observed (Figure 1 and Table S1: 14, 49). On an excursion into the fields of the agricultural area from El Cascajo to El Camote with a local guide, we could not find any barn owls. By contrast, we observed owls on the road heading north from Bellavista to Media Luna (40, 44). However, we could not catch any of these birds, we just saw them flying. We were more successful at finding signs of owls in lava holes (Figure 1 and Table S1: 2, 11, 15, 19, 22–24, 26, 31, 39) or in human-built structures (Figure 1 and Table S1: 1, 3, 5, 8–10, 12, 13, 16, 21, 28, 38, 41–43, 45). These were also the places where we could catch owls (Figure 1, turquoise numbers, Table S1). To our surprise, we rarely observed owls in bigger settlements like Bellavista, a village that we systematically walked several times in early evenings. The best information came from farmers, who reported to us that owls were living on their properties (Figure 1 and Table S1: 4, 8, 9, 10–12, 18, 22–24, 31, 36, 37, 42, 43, 45, 48, 50).

Wherever we encountered barn owls we considered catching them, to fix loggers and obtain foraging data. The condition to do so was that there was not only a chance to catch the birds, but also an equal chance to re-catch the birds. We did not attempt to catch birds when they had chicks, e.g., 2016 in location #8 (Figure 1 and Table S1). The pair at the main house at Miconia (location #1 in Figure 1) was interesting, because it was present from 2014 to at least 2020. We could identify the birds based on their feather morphology and because we had ringed them [8,25]. Thus, we are sure that the same individuals roosted at this location from 2016 to 2020.

We found signs of owls in 11 locations in 2016. We rechecked nine locations in 2019, and found owls in three locations again 3.5 years later (1, 11, 12). Likewise, we found barn owls in 21 locations in 2017. In 2019, eight of these locations were visited again, and barn owls were found in four.

### 3.2. Pellet Collection and Pellet Analysis

We analyzed 523 barn-owl pellets from 20 locations (Table S2). We focused on collecting barn-owl pellets on Santa Cruz at those sites where we also obtained foraging data. Thus, 37 barn-owl pellets were collected in the hut at Finca Nueva York (location #3 in Figure 1), 171 pellets at Finca Miconia (location #1 in Figure 1), and 31 at Royal Palm (location #9 in Figure 1).

Rodent remains were found in 84% of the pellets, while insect remains were found in 83% of the pellets (Table 1). Thus, interestingly, the majority of the pellets (68%) contained remains of both mammals and arthropods. Of the rodents, 86% were mice, while 14% were rats. With respect to biomass, this means that rats, weighing about six times as much as mice [5], made up about the same amount of biomass as mice. If we follow [5], apply his weights for rats (58 g) and mice (10 g) to our numbers, assume a mean weight of 2 g for insects, and consider a biomass of 1% for birds, rodents constituted 89% of the biomass consumed by the barn owls, while insects contributed 10%. We found bird skulls in 6 pellets. We found bones that are most likely from a juvenile chicken in one barn-owl pellet.

### 3.3. Foraging Data

This section is based on 22,735 data points, recorded over 53 days for eight barn owls, amongst them three pairs. Data were collected between 2016 and 2019. Note that these data were not equally distributed between birds. We collected data from 15 days in two different years and seasons (March and November) from two birds, data from seven days for two birds in September/October, data on three days from one bird in March, data on two days in two birds in March, and data on one day in one bird in September. Note that the large difference in tracking time is mainly due to three effects: battery life in relation to sampling interval, logger activity, and repetitive recording. We controlled for a possible influence of recording time and did not observe such an influence. Therefore, data from birds for which we have only one night of recording, still yielded valuable information. In the following subsections, we shall first describe two typical examples of foraging during one night each, one of a single bird (bird C), and one of a pair (N024504, N024505). Then we present general patterns of behavior including observations about all owls on foraging start times, end times, and duration, the size of foraging areas, stop-over points, stop-over durations, and percent of non-motion during night-time foraging.

#### 3.3.1. Typical Examples of Foraging

In 2016 a pair (owls C and D) was encountered in a hut on Finca Nueva York (location #3 in Figure 1), perched on a horizontal bar. These birds were very tame, and could easily be picked from the bar. We caught the birds and fixed a backpack with a logger to each bird. Data for 2.5 days (owl C) and1.5 days (owl D) were recorded before the loggers stopped fixing data because of low batteries.

**Figure 2:**
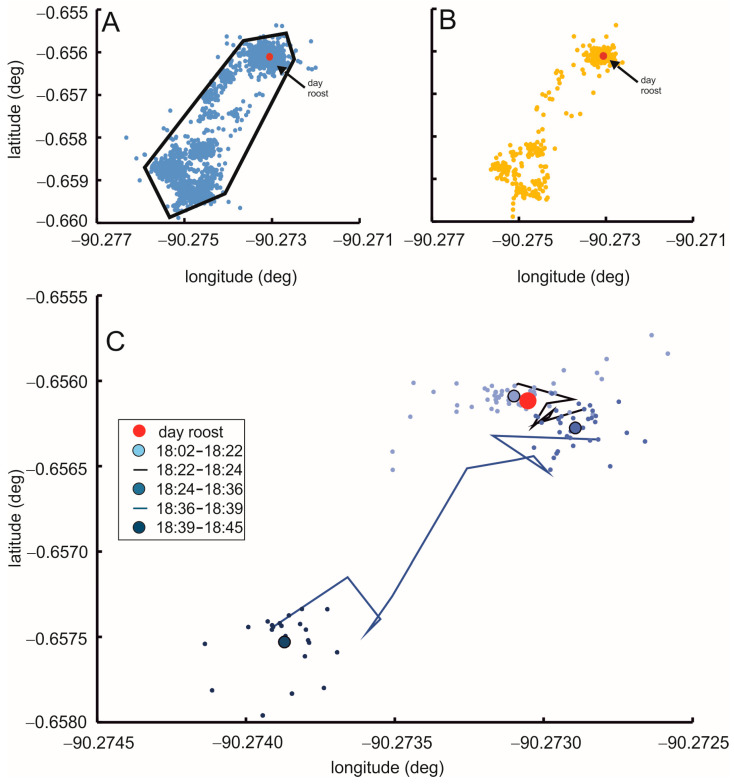
Roosting and foraging of owl C. (**A**) Data recorded with a 20 s time interval from 9:51:46, 1st March to 9:51:10, 2nd March. (**B**) Data sub-sampled to 1 data point per 3 min. (**C**) Time when the owl left the day roost. Lines represent times of movements while small dots signify times of non-motion and large dots with black border represent the mean location during a stopover. Time resolution in (**C**) as in (**A**). In (**A**), the foraging area is marked by the black lines.

All barn owls investigated typically roosted during the day, and started to become active shortly after sunset. A data logger was fitted to owl C around 10:00 local time. Afterwards, the bird was returned to its day roost. Figure 2 shows the data recorded during 24 h. The scatter in the data while the bird rested is due to the spatial resolution of the data logger.

While small movements of the owl are masked by the scatter of the data points, the data suggest that the owl interspersed stop-over points, as revealed by spots of higher dot density, with foraging flights (Figure 2 and Figure S1). The raw data are shown in Figure 2A, while Figure 2B includes the same data, sub-sampled from 1 data point/20 s (Figure 2A) to 1 data point/3 min (Figure 2B). Although some detail is lost, the stop-over points, interspersed between foraging flights, are clearly visible in Figure 2B.

The behavior of the bird during the 24 h recording time may be described as follows: (1) After the bird was returned to the day roost (red dot in Figure 2A–C), it remained there during the day, as is obvious by the scattering of many dots around the location of the day roost (Figure 2A,B, 18:02–18:22 in Figure 2C). (2) At the beginning of the daily foraging trip, the bird first left the roost, but stayed in the vicinity for some time, before it moved on. This movement pattern was reconstructed from the recorded locations as follows: (2a) The locations recorded between 18:02–18:22 scattered around the day roost and were stable with some scatter (big light blue dot with black border within small light blue dots in Figure 2C). The precision of the recording based on 55 data points and measured as standard deviation around the mean in two dimensions was 24 m. Note that within the spatial resolution of the logger data, one may say that the big light blue dot with black border overlaps with the red dot, specifying the location of the day roost. (2b) The center of the recorded locations shifted a short distance between 18:22 and 18:24 (Figure 2C). This is indicated by the black line that denotes the flight trajectory during this time. (2c) The recorded locations scattered about a stable center again between 18:24–18:36 (big blue dot with black border within small blue dots in Figure 2C). This indicated to us that the bird had left the roost at about 18:22, but stayed in the vicinity of the roost until 18:36. Therefore, 18:22 was noted as the start time of the foraging period. In other words, the bird left the day roost about 5 min after sunset that occurred at 18:17 on this day. (3) At 18:36, the bird started to fly away from the roosting area (dark blue line in Figure 2C). (4) The bird reached a stop-over point at 18:39, indicated again by the scattering of the recorded locations around a stable center (big dark blue dot with black border within small dark blue dots in Figure 2C). The bird stayed in this location for about 6 min before it moved on.

The behavior of the bird when it returned to the roost in the morning was similar: the bird first moved close to the roost, and stayed there for some time, before it entered the roost.

About one hour after the bird had left the vicinity of the day roost, at 19:31, it arrived at an agricultural area. The details of the foraging behavior from 19:31 to 23:27 are documented in Figure S1. Briefly, the behavior during this time may be characterized by foraging flights interspersed with stop-overs. The bird stayed the same area until 4:32, when it started to move back to the day roost, thereby pausing three more times (Figure 2A,B). Between 19:31 and 4:32, the bird made two long lasting stops of 2:56 and 1:52 h, respectively (Figure 2A,B).

The end time was at 5:31. In summary, foraging lasted from 18:22 to 5:31 or 11:09 h. During this time, we counted at least 14 stop-overs for this bird and this night. The stops lasted between 6 min and 2:56 h. Roosting time was 8:42 h, resulting in a 78% percent of non-motion. The total foraging area during this night was 0.1 km^2^ (Figure 2A).

The second example that we describe in some detail documents the foraging of a pair that was typically roosting together in a small hut containing an energy station. However, the birds singly also used other day roosts, for example, the entrance to a lava hole not far from the hut.

**Figure 3:**
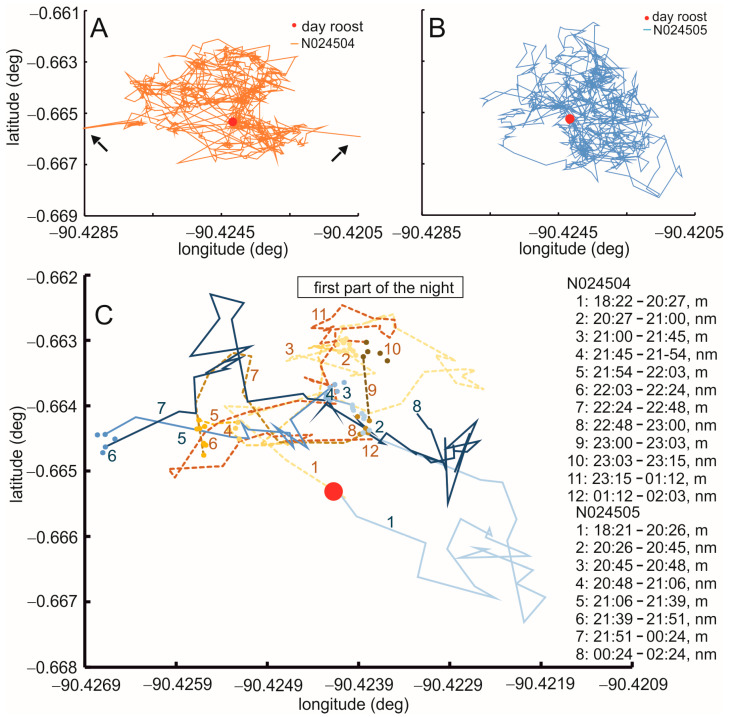
Foraging of a pair of barn owls during seven days of recording. (**A**) Raw data from the male, owl N024504. (**B**) Raw data from the female, owl N024505. (**C**) Details of the foraging paths and stops during the first part of the fifth night of recording. Yellowish-brownish colors and dashed lines: N024504, blueish colors and solid lines: N024505. For all: red dot: location of day roost. Lines represent times of movements (m), while dots signify times of non-motion (nm). Arrows in (A) point to outliers.

Figure 3A shows the raw data for all seven days of recording (26 September to 3 October, 2018) of owl N024504, a male. The foraging area of this bird in these seven days had a size of 0.37 km^2^. The foraging area scattered around the day roost (red dot in Figure 3) with a bias to the west. The foraging area covered mainly meadows. Note the spots of denser lines that indicate stop-overs. A similar behavior was observed in the female, owl N024505. This bird foraged in an area of a similar size, 0.44 km^2^, shifted a bit to the east of the day roost (Figure 3B). Figure 3A shows two examples of outliers (see arrows in Figure 3A). Note that the lines leading to these points appear as spikes and clearly differ from the bulk of the other points.

During the fifth night of recording, the birds foraged independently, as is detailed in Figure 3C and Figure S2. Independent foraging did not only occur in this night, but also in the other nights recorded. Moreover, the other two pairs of owls from which we have data foraged independently. For example, the birds left the day roosts in different directions (start times N024504: 18:22, N024505: 18:21; ones in Figure 3C). Start times were 16 and 15 min after sunset, respectively. The first stop of both birds was at a similar time (twos in Figure 3C). Owl N024504 stayed, however, much longer in the stop-over location than owl N024505 (33 vs. 19 min) before it moved on (threes in Figure 3C). Owl N024504 had four more stops until midnight, all in a small area (yellowish-brownish #s 4, 6, 8, 10). By contrast, owl N024505 had only two more stops during this time, also in a small foraging area (blueish #s 4 and 6). The owls then stayed in one place for some time (owl N024504: 1:12–2:03; owl N024505: 0:24–2:24). In the second part of the night, this pattern continued (see Figure S2 for data and explanation).

The birds returned to the day roosts shortly before sunrise at 5:48 (owl N024504 at 5:34, owl N024505 at 5:30). Foraging times, thus, were 11:12 and 11:09 h, respectively. While owls N024504 and N024505 had a similar number of stops as owl C (compare Figure 3 with Figure 2), and foraging times were similar, the former birds spent more times flying than the latter bird (N024504: 68%, N024505: 69%; owl C: 22% of foraging time, respectively).

#### 3.3.2. General Patterns of Foraging

**Figure 4:**
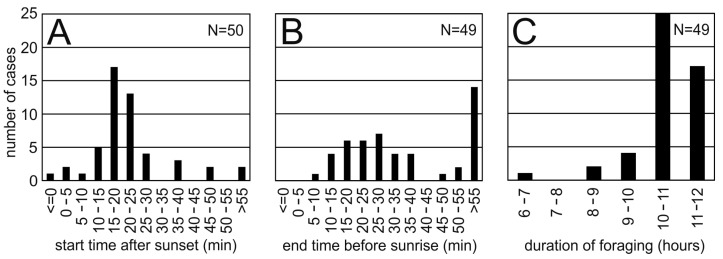
Start time, end time, and duration of night-time foraging. (**A**) Distribution of start times relative to sunset. (**B**) Distribution of end times relative to sunrise. (**C**) Duration of foraging.

The owls started night-time foraging between 0 and 78 min after sunset (Figure 4A). This corresponded to absolute start times between 18:02 and 19:43 local time. The data were clearly scattered narrowly around the median value of 20 min. A Mann-Whitney U-test did not show significant differences in start times of males and females (number of male data points: 28; number of female data points: 24; U = 301.5, z-score = 0.62409; *p*-value = 0.53526).

Likewise, night-time foraging ended shortly before sunrise (median: 31 min). However, the data were much less concentrated, and there was a long tail towards an earlier return to the day roost (Figure 4B). Indeed, in 28.5% of the cases (14 out of 49), foraging ended more than an hour before sunrise. The earliest end time was at 0:54, or almost 5 h before sunrise, while the latest end time was 6:00 local time. A Mann-Whitney U-test did not show difference in end times of males and females (number of male data points: 26; number of female data points: 23; U = 275.5, z-score = 0.46077; *p*-value = 0.64552).

The distribution of the foraging durations was also concentrated around a narrow maximum with a median of 10:51 h (Figure 4C). The maximum foraging time was 11:42 h, only slightly shorter than the time between sunset and sunrise (about 11:50 h). The early return becomes also obvious in the distribution of foraging times as the shortest foraging duration (6:34 h). A Mann-Whitney U-test did not show difference in foraging durations of males and females (number of male data points: 26; number of female data points: 23; U = 289, z-score = 0.19032; *p*-value = 0.8493).

The foraging areas were calculated from all data available for a bird. Based on eight data points, the median was 0.28 km^2^, with the lower quartile at 0.18 km^2^ and the upper quartile at 0.32 km^2^.

**Figure 5:**
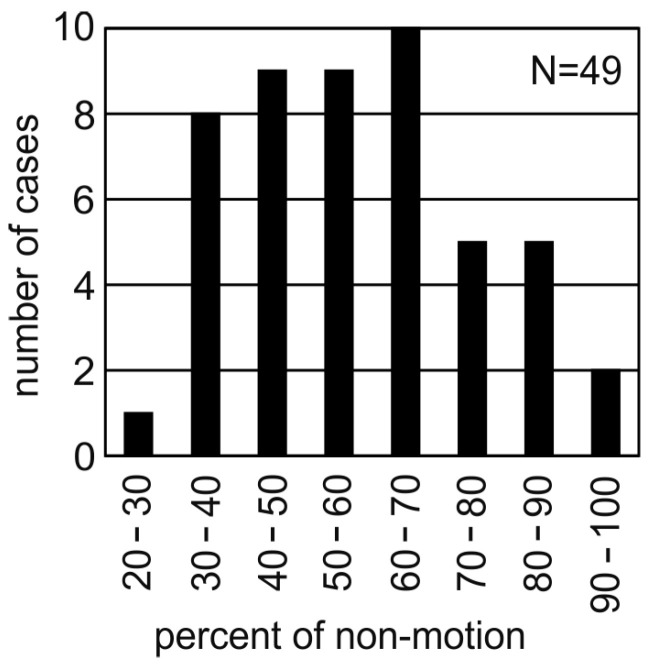
Time spent in one place during night-time foraging.

During night-time foraging, the birds spent part of the time moving from place to place, but most of the time they stayed in one place (median: 56%, lower quartile: 44%, upper quartile: 69.5% of the time) (Figure 5). The maximum time spent in one place by a bird during one night while foraging was 98%, the minimum time was 22%. Interestingly, females spent less time not moving than males (Mann-Whitney U-test (number of male data points: 26; number of female data points: 23; U = 160, z-score = 2.77463; *p*-value = 0.0056)).

What we call “staying in one place” here is a complex mix of behaviors, including searching for prey from perches, eating prey, and resting. Our data do not allow to discriminate between these possibilities. The data specifically do not allow to determine when the bird caught prey and moved to a location where it ate the prey and how long this lasted.

Overall, in the 49 nights for which complete data are available, we counted 1 to 14 stops per night with a median of 8. This number, however, should be taken with some caution as mentioned above, because we may have missed some short stops, and may have lumped some shorter stops into one longer stop. A Mann-Whitney U-test did not show difference in the number of stops per night of males and females (number of male data points: 27; number of female data points: 23; U = 275.5, z-score = 0.67155; *p*-value = 0.50286).

### 3.4. Estimation of the Abundance of Barn Owls in the Agricultural Area of Santa Cruz

**Figure 6:**
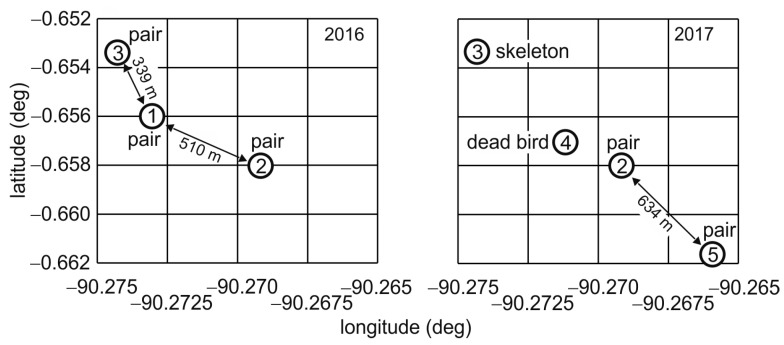
Barn-owl density. Note that locations #1–3 correspond to sites 3, 2 and 7, respectively, in Figure 1, while locations #4 and #5 correspond to sites 25 and 19 in Figure 1.

The small foraging areas suggested that barn-owl density may be high on Santa Cruz (see also [5]). The few data that we have are consistent with such a claim. For example, in 2016 we encountered three pairs very close to each other in the eastern part of the agricultural area, characterized by meadows and woodland (Figure 2 and Figure 6). The pair at location #1 (Figure 6) was caught and foraging data were obtained (see owl C above). A year later, owls were no longer found at location #1, but rather at location #2 and a new location, location #5 (Figure 6). There was also a skeleton of a dead bird near location #3 and a dead bird in a water tank at location #4. We did not find more barn owls close by.

In other areas we observed a similar density, for example, in 2019 in Finca Miconia. Here, a pair was roosting under the main house (site 1 in Figure 1), while a single bird was encountered in a shed about 200 m to the north-west (site 42 in Figure 1), and another bird was found in the old house (site 43 in Figure 1), about 1100 m from the main house.

## 4. Discussion

### 4.1. Owl Abundance

Our data confirm the continued presence of *T. f. punctatissima* in the agricultural area of Santa Cruz Island and offer valuable insights into space use and potential local abundance. GPS tracking and field observations indicate that foraging areas in this zone are relatively small (median 0.28 km^2^), much smaller than those reported in temperate regions such as Europe—where densities range from 2–4 pairs per 10 km^2^ [28]—or in Argentina [29]. These smaller home ranges may indicate the capacity for higher local densities in Galápagos, possibly due to habitat structure or introduced prey (*Rattus* spp.) availability, but this cannot be extrapolated to pristine islands. Published estimates of total barn owl abundance in the archipelago vary widely. For example, Swash and Still [1] mention an estimate of 9000 barn owls in the Galápagos Archipelago, while de Groot [5] reports approximately 8500 pairs, primarily concentrated on the larger islands (see also [2,30]). However, the methodology behind these estimates is not well documented, and their current relevance is uncertain. Our own data, though limited in scope, provide important evidence that can contribute to refining future assessments. This insecurity and the threats that have arisen lately (see below) have resulted in classifying the Galápagos barn owl as endangered (EN—C2a(i)) in the IUCN Ecuador Threatened Red List in 2019). However, available databases, including our own, are not large enough to make firm conclusions on trends. Nonetheless, emerging threats such as increased vehicle collisions, not accounted for in earlier studies, justify precaution [23,31,32,33]. Many more data are necessary before a definitive statement on owl abundance in the agricultural area of Santa Cruz can be made. Furthermore, population studies outside the agricultural area and on non-inhabited islands, such as Santiago, seem necessary to investigate barn-owl densities of the entire archipelago.

### 4.2. Owl Diet

When discussing the diet of barn owls, it is important to consider that barn owls are opportunistic predators that will consume any small animal available. Although one may be concerned about a sampling bias, because many of our data come from only a few collection sites, and the different studies were not based on methodological standardization, our dietary data are by and large consistent with the results presented in [5] and in [19]. However, the number of birds in our data is significantly less than those reported in [19]: 1% in our sample vs. 9.7% in the sample in [19]. De Groot [5] reported 1% of bird items in the pellets he analyzed. This is consistent with our data. Although local farmers claim that barn owls prey on chickens and cite this as a reason to kill them, we found chicken remains in only 1 out of 523 pellets, and none were reported in [5]. Since we collected many pellets in or near farms with chickens, we argue that the small number of remains of birds, especially those of chickens, is not a consequence of a sampling bias. Our findings, together with those in [5], do not support the claim that Galápagos barn owls prey on domestic birds. One possible reason for the misinterpretation of laypersons may be that they do not distinguish between the two owl subspecies. It is known that the Galápagos short-eared owl consumes more birds than the Galápagos barn owl [5,19].

The proportion of small mammals in the diet of barn owls is similar across all three data sets, our own and those reported in [5] and in [19], with values consistently around 85%. These results align with data reported from other continents [11,28]. Galápagos barn owls include a substantial proportion of insects in their diet. In de Groot’s [5] sample, insects accounted for approximately 12%, which we regard as consistent with the 10% found in our data. These values are higher than those reported from Europe [30]. Overall, a comparison with the data of de Groot [5] and Abs et al. [19] suggests that the diet of barn owls in the Galápagos has remained stable over the past 40–60 years.

Note that there is also a conservation dilemma in that recently eradication efforts of rats and mice has increased. These invasive species have become the main prey for the owls. It is unclear how owls will react and to what food they will shift if rats and mice are no longer available, as is the case in Pinta and Floreana. However, barn owls do not inhabit Pinta and Floreana, and it is not feasible to eradicate all rodents in Santa Cruz. Therefore, this dilemma has to be studied in the other owl species living on Galápagos, the short-eared owl [4].

### 4.3. Foraging Movements

Data from GPS loggers provided important insights into the foraging behavior of Galápagos barn owls. Such data have never been recorded before in Galápagos barn owls. First, sub-sampling high resolution temporal data (1 datum/20 s) to one point every 3 min did not affect the main conclusions, consistent with findings in [34]. Second, we could not detect any sign that the loggers impaired foraging behavior. We could also not detect signs of unnatural behavior in the first hours after mounting the backpack. This is consistent with observations by others that conducted similar experiments [26,29,34,35,36]. Thus, we conclude that our logger data showed the natural behavior of the owls. Third, despite the relatively small sample size, several consistent patterns were observed. Foraging areas were markedly smaller as those reported in Europe, the USA, Argentina, or Israel [11,28,29,34], and would fall into the “closed, very small local” category as proposed in [34]. The size of the foraging areas did not change much across nights and over one-week intervals. In one pair, for which we have data separated by 32 months, the size of the foraging area showed no substantial changes over time. Night-time foraging was characterized by alternating periods of flight and stop-overs, with longer stationary phases around midnight, a pattern observed in all tagged birds. Similar activity cycles have been found in laboratory conditions under artificial light-dark regimes [37] and in the field by others [5,38]. Median percent of non-motion (56%) was lower than reported in [5] (72%). Males and females hunted independently, typically differing in timing of departure and return, stopover locations, and durations of non-motion. This behavior has also been reported outside of Galápagos [11]. Independent hunting is not restricted to sex, but seems to be a personality trait [39]. Start and end times in Galápagos barn owls were similar to those observed in Europe (observation by HW), but slightly closer to sunset (20 min). This indicates that the birds use a certain light level to start (and end) night-time foraging [37]. It might even reflect the faster sunset and sunrise close to the equator compared to Europe. Unfortunately, the end of foraging activity coincides with increased car traffic to the Itabaca Channel from Puerto Ayora, leading to high rates of barn owl roadkills along this route [23].

### 4.4. Outlook

We regard the situation for the barn owl on Galápagos as labile because the subspecies faces several threats that may increase in the future. External causes of mortality, such as vehicle collision, entangling in barbed-wire fences [31], or intentional killing, may create instability that cannot be offset by increased reproductive output, unlike in Europe, where owl populations fluctuate but can compensate for losses during bad years with larger broods in years with abundant prey (see below). One of the main threats is road mortality along the main road from Puerto Ayora to Itabaca Channel. Car traffic has increased over the past decades on the islands, and will likely continue to increase. Although warning signs are placed to alert drivers to the risk of collision with birds, our observations suggest that many vehicles exceed speed limits. Barn owls have a slower reaction time at low-light level [23,37] and are especially vulnerable when returning to their roosts at dawn. Another, equally important, threat to barn owls is the unintentional and intentional killing by farmers. This occurs for three main reasons. First, barn owls may die from poisoning. Chemical control of rodents has intensified in recent years. Since rodents constitute the primary prey of barn owls, the increased chemical control may have a significant impact on the barn owl population. Second, farmers claim that barn owls prey on their chickens. While isolated incidents cannot be ruled out, neither our data nor the data of de Groot [5] support this as a common occurrence. Third, a cultural belief persists that barn owls bring misfortune or death, an association found globally and also reported in the Galápagos. Other threats such as the effect of pollutants, diseases, and energy production (wind parks) have not been scrutinized [31]. Our concerns are further aggravated by the fact that barn owls in the Galápagos produce few offspring per brood. Our observations, alongside those reported in [5] and in [19], show that barn owl nests in Galápagos typically contain only one or two chicks, in contrast to the 4–7 typically reported in Europe [11,28].

Thus, action seems necessary to improve the conservation of this unique barn owl subspecies. Moreover, barn owls provide valuable ecosystem services by controlling rodent populations without harming livestock. A single owl consumes roughly two rodents per night [11,28]. Therefore, a pair of owls can eliminate over 1500 rodents annually. During breeding, this number can surpass 2000, with some estimates going far beyond this number. This ecosystem service should compensate for the occasional loss of a chicken. A program led by Conservation International (Sandra Garcia) is fostering collaborations with farmers to install nest boxes on farms. Some farmers have already recognized the advantages that barn owls represent. These farmers accept and protect barn owls, amongst them many of the people who helped us in this study. It is important to promote a positive image of barn owls in the Galápagos Islands as a beneficial and ecologically valuable species, helping communities particularly farmers by coexisting with this endemic predator. Based on the observations reported here, we specifically recommend to better enforce the speed limit on the road from Puerto Ayora to Itabaca Channel, the install of nest boxes in as many farms as possible, and, at the same time, educate locals to promote the recognition of the positive role of the barn owl in this World Heritage ecosystem.

## 5. Conclusions

Our novel insights into the foraging behavior of Galápagos barn owls are consistent with observations from other regions, specifically Europe [11,26,28]. Despite many threats, our data suggest that barn owls continue to be present in the agricultural area on Santa Cruz. Not much is known, however, about the barn owl population outside this area or the changes in population size in the last years. Because of the many threats mentioned, we regard the situation for the subspecies as labile.

While conditions at certain locations have been stable with the same pair occupying a location for several years, others have been more dynamic, influenced by both weather and human activity. For example, a drought in 2016–2017 led to the removal of cattle from some farms, resulting in the loss of a roost for one pair of owls. We have encountered many sites where owls had previously been present but later abandoned. It remains uncertain how barn owls will cope with increasing pressures from human activities and climate change in the years ahead. The listing as endangered mentioned above should prompt authorities to develop a management plan to improve conservation of the Galápagos barn owl in the future, as indicated in the last section.

## Figures and Tables

**Table 1:** Pellets analyzed.

Number of Pellets	Rodents Only	Birds	Rodents and Arthropods	Arthropods Only
523 (100%)	16%	1%	68%	15%

## Data Availability

Foraging data are available at Movebank: https://www.movebank.org/cms/webapp?gwt_fragment=page=search_map (accessed on 29 July 2025).

## Supplementary Materials

The following supporting information can be downloaded at: https://www.mdpi.com/article/10.3390/ani15152283/s1. Figure S1: foraging behavior of a single bird; Figure S2: Example of foraging of a pair of barn owls; Table S1: Locations where signs of barn owls were detected; Table S2: List of collection sites of the pellets analyzed.
