# Supplementary material for: Abundance, Diet and Foraging of Galápagos Barn Owls (Tyto furcata punctatissima)
Source: Animals (Basel). 2025 Aug 5;15(15):2283. doi: 10.3390/ani15152283 (PMC12345479; doi:10.3390/ani15152283)
Supplement: Supplementary file 1 [file animals-15-02283-s001.zip › Figure S1 and description of behavior.pdf]

Figure S1: foraging behavior of a single bird

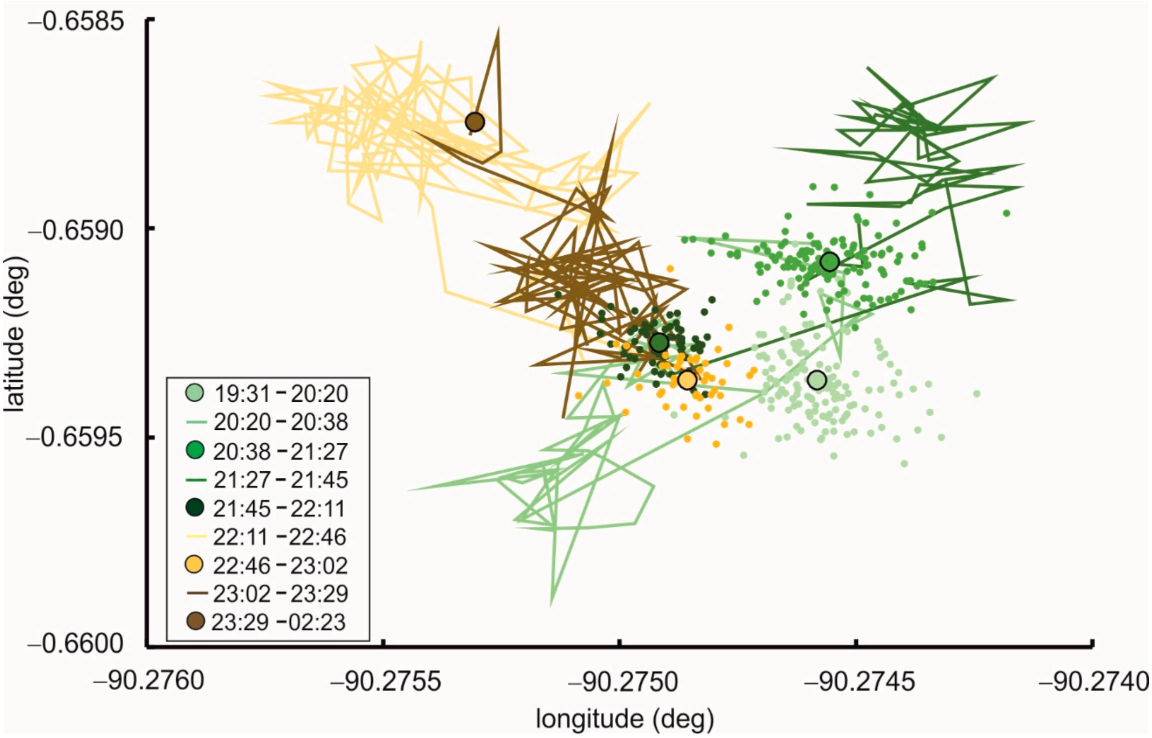

Figure legend and explanation. Foraging behavior of owl C recorded with a 20 sec time interval from 19:31:46, March 1st to 23:27, March 1st, 2016. 1) The bird stayed in one location from 19:31 to 20:20. 2) The bird then foraged in a more south-westerly direction before it flew to the north-east (20:20 to 20:38). 3) The next stop was only a short distance to the north-east of the first stop (20:38 to 21:27). 4) From there the bird foraged further to the north east (21:27 to 21:45), before it stopped once more (5), this time a bit west of the preceding stop (21:45 to 22:11). 6) After this time, the bird scrutinized a region to the north west (22:11 to 22:46), and came to a next stop (7) very close to the last stop (22:46 to 23:02). 8) For the next foraging sequence (23:02-23:29), the bird stayed close by. It then settled in for a long stop-over (2:56 hours) in the area to the north-west that it had scrutinized before. The big dark brown dot with black border shows the mean of the positions recorded during this stop-over. The bird stayed in this area until 4:32, when it started to move back to the day roost, as mentioned also in the main text.
