# Supplementary material for: Abundance, Diet and Foraging of Galápagos Barn Owls (Tyto furcata punctatissima)
Source: Animals (Basel). 2025 Aug 5;15(15):2283. doi: 10.3390/ani15152283 (PMC12345479; doi:10.3390/ani15152283)
Supplement: Supplementary file 1 [file animals-15-02283-s001.zip › Figure S2 Foraging of a pair of barn owls during 7 days of recording.pdf]

Figure S2 Example of foraging of a pair of barn owls

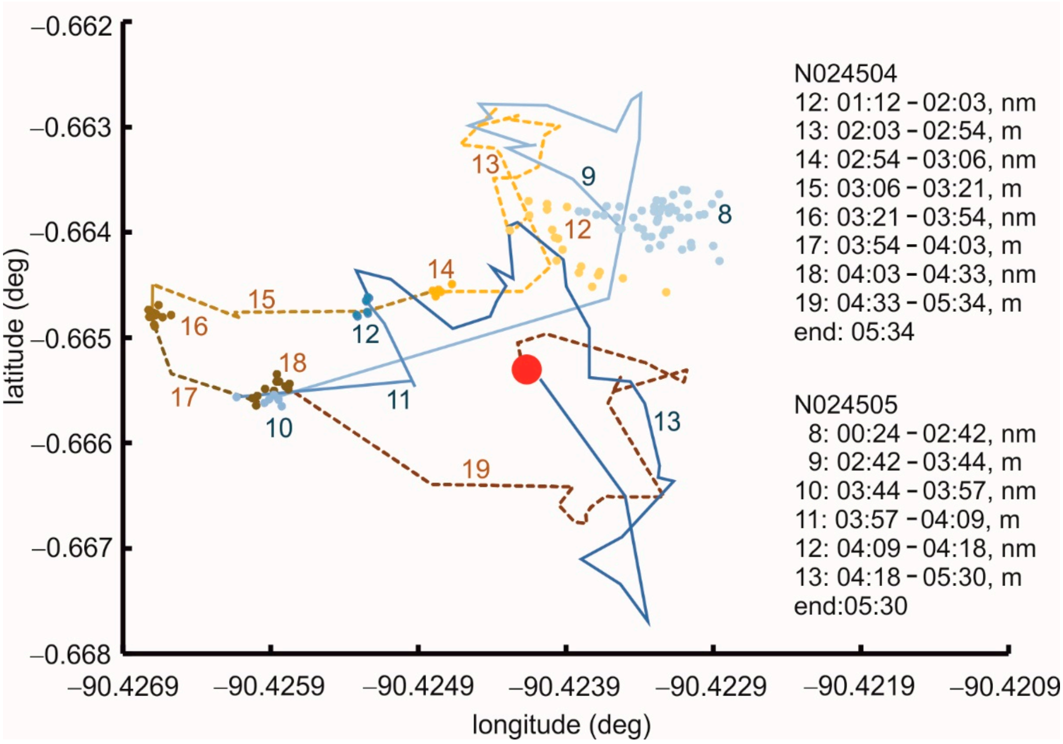

Figure legend and explanation: Data recording lasted 7 days. This figure shows as short, but typical sequence, of the foraging paths and stops during the second half of fifth night of recording. Yellowish-brownish colors and dashed lines: N024504, blueish colors and solid lines: N024505. For all: red dot: location of day roost. Lines represent times of movements (m), while dots signify times of non-motion (nm). Owl N024504 had four stops after midnight (yellowish-brownish #s 12, 14, 16, 18), while owl N024505 remained in one location for three times (blueish #s 8, 10, 12). Each of the birds had a longer period of non-motion shortly after midnight (owl N024504: #12; owl N024505: #8). Note that the birds hunted independently.
