# Supplementary material for: Abundance, Diet and Foraging of Galápagos Barn Owls (Tyto furcata punctatissima)
Source: Animals (Basel). 2025 Aug 5;15(15):2283. doi: 10.3390/ani15152283 (PMC12345479; doi:10.3390/ani15152283)
Supplement: Supplementary file 1 [file animals-15-02283-s001.zip › Table S2 Wagneretal.pdf]

Table S2: List of collection sites of the pellets analyzed

**No.**	**Location**	**Number***
	Sum	523
1	Finca Miconia	171
2	Finca Nueva York, lava tunnel 1	75
3	Finca Nueva York, hut	37
4	Finca Lava Java	19
5	Charles-Darwin Station	6
7	Finca Fernando Ortega	4
8	Restaurante Narwal	13
9	Royal Palm	31
10	Galápagos Magic	16
12	Las Primicias	4
13	Propiedad de Julio Inga	1
14	Garrapatero	4
15	Túneles de amor (Bellavista)	18
16	Ecolodge Galapagos	2
18	Finca Miramar 2	5
20	El Occidente	16
23	Finca Kastdalen (Miramar)	21
24	Highland View	39
	Two unknown locations	41

* Numbers correspond to numbers in Figure 1.
