# Supplementary material for: Abundance, Diet and Foraging of Galápagos Barn Owls (Tyto furcata punctatissima)
Source: Animals (Basel). 2025 Aug 5;15(15):2283. doi: 10.3390/ani15152283 (PMC12345479; doi:10.3390/ani15152283)
Supplement: Supplementary file 1 [file animals-15-02283-s001.zip › Table_S1.pdf]

Table S1: Locations where signs of barn owls were detected

No.	location	2014	2016	2017	2018	2019	2020	2021	2022
1	Finca Miconia near house and pool	1 m*, 1 f*	1m, 1f, 2 ba-bies, P	P*, T*, 1m: gr., 1f: red	T, P, 1m (gr. == N024501, 1f (red == N024502)	N024501, N024502, T, P	1m, 1f		
2	Finca Nueva York, lava tunel 1		1m, 1f	P		--			
3	Finca Nueva York, hut		1 m, 1f, T, P	P	--				
4	Lava Java		1 m, 1f, 1juv, P	P	--				
5	Charles-Darwin Station		1f		P	P			
6	El Trapiche		1 m	--		--			
7	Finca Ortega		1m, 1f	1ex*, †	1ex, P	P			
8	Narwhal		1m 1f, 2 babies, P	1m, 1f, P	1ex, P	--			
9	Royal Palm		1f, P	1m, T	1f				
10	Galapagos Magic		P	P	1m, P	P			
11	Manzanillo		1f, P	1m, 1f, P	P, T, 1m (N024503)	P, 1f (N024510), N024503	1m, 1f		1 ex
12	Primicias		1f, 1m, P	1m, 1f, P	T, P,1m (N024504), 1f (N024505),	P, 1m, 1f (ring re-cognized: N024505)			
13	Julio Inga property		P						
14	Garrapatero			P					
15	Tuneles de Amor, Bellavista			1ex, P		P			
16	Ecolodge Galapagos			P					
17	Las Grietas			1m	--	--			
18	Miramar 2		--	1f, bl. ring	1m, 1f, P	--			
19	Finca Nueva York, lava tunel 3			1m, 1f, P		1f			
20	El Occidente			1m, P					
21	Galapagos Lodge			1m					
22	Jonathan Caisaguano farm			1m, 1f	--				
23	Kastdalen			1f, 2ex, P					
24	Highland View (several tunnels)		--	1f, P	1ex, P		1		
25	Nueva York, water tank			1 ex, †					
26	Finca Nueva York, lava tunel 2			1m, 1f, P	P				
27	Granillo rojo			1m, call*					
28	Pajaro Brujo		--	P	P				

29	km 9			1f, call					
30	km 8			1f, call					
31	El Chato 2			P	P				
32	Granillo negro			1m, 1f, call					
33	km 8				1 ex, †				
34	km 8 +				1 ex, †				
35	km 32				1 ex, †				
36	Pikaya				1m, 1f				
37	Mariposa	1			1m, 1f	1m (N024511), 1f (N024509)			
38	Universidad Central Ecuador				P	--			
39	Tunels de Puerto Ayora				1m, 1f, P	P			
40	Road to Media Luna			--	1 ex				
41	Tomas de Berlanga					1ex			
42	Finca Miconia, cattle shelter					1 m (N024508)			
43	Finca Miconia, old house			--		1 f (N024507)			
44	Bellavista North					P			
45	Finca Schiess					1 f	1ex		1ex
46	Km 34					1 ex, †			
47	Southwest Bellavista							1f	
48	Property Cruz							1m, 1ex	
49	Road to Cascajo						1ex		
50	Javier Moreno								2ex
51	Alba Lava tunnels								2ex

* m=male, f=female, ex=exemplar, T=Logger fixed and data collected, P=Pellets present, F=Feathers present, Nxxxxxx=Ring number, --: nothing observed, gr.=green ring, bl=blue ring, †=dead, call=calling
